# Burden in caregivers of primary care patients with dementia: influence of neuropsychiatric symptoms according to disease stage (NeDEM project)

**DOI:** 10.1186/s12877-023-04234-0

**Published:** 2023-08-29

**Authors:** Victoria García-Martín, M Canto de Hoyos-Alonso, Rosalía Delgado-Puebla, Gloria Ariza-Cardiel, Isabel del Cura-González

**Affiliations:** 1 Epidemiology and Public Health, Universidad Rey Juan Carlos (Rey Juan Carlos University), Madrid, Spain; 2 Pedro Laín Entralgo Health Care Center, Primary Care Management, Madrid Health Service, Alcorcón, Madrid, Spain; 3 Network for Research on Chronicity, Primary Care and Health Promotion (RICAPPS), Madrid, Spain; 4 Primary Care Management, Horta Health Care Center, Catalonia Health Service, Barcelona, Catalonia Spain; 5 Family and Community Medicine Teaching Unit Oeste, Primary Care Management, Madrid Health Service, Móstoles, Madrid, Spain; 6 Research Unit, Primary Care Management, Madrid Health Service, Madrid, Spain; 7 Department of Medical Specialties and Public Health, Universidad Rey Juan Carlos (Rey Juan Carlos University), Alcorcón, Madrid, Spain; 8 Ageing Research Center, Karolinska Institutet and Stockholm University, Stockholm, Sweden; 9 Instituto Investigación Sanitaria Gregorio Marañón IiSGM, Madrid, Spain

**Keywords:** Dementia, Caregiver burden, Neuropsychiatric symptoms, Behavioral and psychological symptoms of dementia, Zarit burden interview, Neuropsychiatric inventory caregiver distress scale, Neuropsychiatric inventory, Global deterioration scale, Primary care

## Abstract

**Background:**

Caregiver burden is related to personal factors and patient characteristics and is greater when neuropsychiatric symptoms (NPSs) are present. Objective: Estimate the prevalence of burden among caregivers of dementia patients and its association with NPSs and identify NPSs causing greater caregiver distress according to dementia stage.

**Methods:**

A cross-sectional observational study in caregivers of noninstitutionalized dementia patients was conducted. Caregiver variables were sociodemographic, time of care, NPS-associated distress based on the Neuropsychiatric Inventory Caregiver Distress Scale (NPI-D) and burden based on the Zarit Burden Interview (ZBI). Patient variables were time since disease onset, Global Deterioration Scale (GDS) disease stage, functional assessment and NPS presence and intensity according to the Neuropsychiatric Inventory (NPI). The mean ZBI score, prevalence of burden and NPI-D score with 95% CIs at each dementia stage were estimated. Factors associated with burden were identified by multivariate analysis.

**Results:**

Of the 125 caregivers included, 77.6% were women, with a mean age of 60.7 (± 14.3) years; 78.4% (95%CI: 71.0; 86.0) experienced burden. The mean ZBI score was 12.3 (95%CI: 11.6; 12.9) and increased according to NPS number (p = 0.042). The NPSs causing the most burden were disinhibition (93.5%), irritability (87.3%) and agitation (86.1%). Agitation, apathy, and sleep disorders were the NPSs generating the greatest overall caregiver distress; depression (max NPI-D 1.9), hyperactivity (max NPI-D 2.1), and psychosis symptoms (max NPI-D 1.6) generated the greatest distress at stage GDS 3, stages GDS 4–5, and stages GDS 6–7, respectively. The NPI score (OR = 1.0, 95%CI 1.0; 1.1), intensity of irritability (OR = 1.2, 95%CI 1.0; 1.6), disinhibition (OR = 2.6, 95%CI 1.1; 5.8) and hyperactivity subsyndrome (OR = 1.1, 95%CI 1.0; 1.2) were associated with caregiver burden. Other associated factors were female gender (OR = 6.0, 95%CI 1.6; 22.8), ≥ 8 h daily care (OR = 5.6, 95%CI 1.4; 22.8), working outside the home (OR = 7.6, 95%CI 1.8; 31.8), living with the patient (OR = 4.5, 95%CI 1.1; 19.6), kinship (OR = 5.4, 95%CI 1.0; 28.2) and lower patient education (OR = 8.3, 95%CI 2.3; 30.3).

**Conclusions:**

The burden on caregivers of dementia patients is high and associated with NPS presence and intensity. Disinhibition and irritability caused the highest burden. Depression, hyperactivity and psychosis produce more distress in mild, mild-moderate and severe dementia, respectively.

**Supplementary Information:**

The online version contains supplementary material available at 10.1186/s12877-023-04234-0.

## Background

The prevalence of dementia or major neurocognitive disorder varies according to age, sex and geographic location [1]. In 2019, more than 57 million people worldwide had dementia, and it is estimated that this number will increase to 152 million by 2050 [2], which will impact patients as well as their caregivers, families and society. The care and treatment of patients with dementia represent a significant financial burden [3, 4] that varies by country [5]. In Europe, the estimated average annual cost of a patient with dementia is €32,506.73 [6], and this amount increases with greater dementia severity or when patients are institutionalized [4, 5].

Different social and cultural aspects determine the place where care is provided (at home or in health care institutions) and the individual in charge of providing the care (family members or professional caregivers) [7]. Typically, those responsible for care are women (wives or daughters) who, in addition, tend to assume a greater burden than men [8–10]. Burden is related to caregiver factors, such as gender [11], hours of care or kinship [12] and patient characteristics, and is greater when neuropsychiatric symptoms (NPSs) are present [13, 14].

NPSs or behavioral and psychological symptoms of dementia (BPSD) appear frequently throughout the disease [15]; they worsen the prognosis [16] and are a frequent reason for institutionalization [17–19]. They are associated with depressive symptoms, decreased quality of life and caregiver burden [20–23]. The validated scales used to measure burden in caregivers of patients with dementia include the Zarit Burden Interview, Caregiver Burden Interview, Screen for Caregiver Burden and the Burden Scale for Family Caregivers [24]. Caregiver burden can also be measured using the Neuropsychiatric Inventory Caregiver Distress Scale [25]. This scale specifically measures the burden or distress caused to the caregiver by the different NPSs exhibited by the patient.

The objective of this study was to estimate the prevalence of burden in caregivers of noninstitutionalized patients with dementia, to analyse its association with NPSs or groups of symptoms (subsyndromes) and to describe which NPSs result in the most distress in caregivers according to the different dementia stages.

## Methods

### Study design, setting and participants

This was a cross-sectional, descriptive, observational study in primary care. This study included caregivers of patients with dementia treated in the last year at health centers of the urban municipalities of Alcorcón and Villaviciosa de Odón (Madrid, Spain) who were neither deceased nor institutionalized during the study (1 November 2015 to 26 February 2016). For the selection of patients, patients of all ages with a previous diagnosis of dementia identified in the electronic health records (EHRs) of the Community of Madrid (AP-Madrid) with the code P70 according to the International Classification of Primary Care (ICPC) and/or with specific treatment for it, anticholinesterase drugs (ATC code: N06D) and/or memantine (ATC code: N06DX01) were included. All participants provided signed, informed consent. Caregivers who had difficulties with the Spanish language were excluded from the interviews. The data were collected by reviewing the EHRs of the patients and interviews with the caregivers that included different validated scales to analyse NPS [26] and caregiver distress [27]. In cases where one patient had more than one caregiver, only the primary caregiver was considered for the study. When the same caregiver cared for more than one patient, an interview was conducted for each patient. According to these criteria, 125 caregivers of 129 patients with dementia were included. The clinical and epidemiological characteristics of the patients included here are described in a previous study [28].

The results of this study were reported according to the STROBE recommendations [29].

### Variables and assessment instruments

The sociodemographic variables collected for caregivers were age, gender, highest educational level completed, employment status, marital status, kinship to the patient, and cohabitation status with the patient. Variables related to care were also collected, namely, time of care in months/years, in days per week and in hours per day and the levels of burden and distress caused by NPSs.

Caregiver burden was measured using the short Zarit Burden Interview (ZBI) in dementia [30], which explores four items related to self-care, stress and burden scored from 1 (never) to 5 (almost always), for a total score ranging from a minimum of 4 points to a maximum of 20 points. It is a screening test to diagnose burden in caregivers of patients with dementia, with high sensitivity and specificity (98.5% and 93.9%, respectively). It is shorter than the original 22-item Zarit test [31] and validated for the Spanish population [32]. The short ZBI in dementia considers burden to be present when the total score is ≥ 10 points and absent when the score is < 10 points. In caregivers who care for more than one patient, the burden caused by each patient was measured separately.

The distress in caregivers caused by the patients’ NPSs was measured using the Neuropsychiatric Inventory Caregiver Distress Scale (NPI-D) [25, 27]. This scale is scored from 0 to 5 points, with 0 = not at all distressing, 1 = minimally distressing, 2 = mildly distressing, 3 = moderately distressing, 4 = severely distressing and 5 = very severely or extremely distressing. The total score, which ranges from 0 to 60, is the sum of the distress scores for each symptom. Distress was classified according to Kaufer et al. (1998) [25] as low (NPI-D score 0–1: not at all to minimally distressing), medium (NPI-D score 2–3: mild to moderately distressing) or high (NPI-D score 4–5: severely to extremely distressing).

The variable collected on patients was functional independence using the Barthel Index[33] classified according to the dependence levels established by Shah et al. [34]; time since onset of dementia and dementia stage according to the Reisberg Global Deterioration Scale (GDS) [35], where GDS3 corresponds to mild cognitive impairment and GDS7 corresponds to very severe cognitive impairment; presence and intensity of the NPSs as measured by the Neuropsychiatric Inventory (NPI) scale [26, 36]; and specific treatment for dementia (anticholinesterase and/or memantine) and treatment to alleviate NPSs (neuroleptics, antidepressants and benzodiazepines).

The NPI scale explores 12 NPSs: delusions, hallucinations, aggressiveness, depression, anxiety, elation/euphoria, agitation/aggression, apathy/indifference, disinhibition, irritability/lability, aberrant motor behavior, sleep behavior and appetite/eating behavior. The total NPI score (0 to 144) is calculated by adding the intensity of these twelve symptoms, obtained from the product of the frequency (score of 1 to 4) and severity (score of 1 to 3) of each.

The symptoms were grouped into four subsyndromes according to the classification of Aalten et al. (2007) [37]: “hyperactivity” (agitation/aggression, disinhibition, irritability/lability, aberrant motor behavior and elation/euphoria); “psychosis” (hallucinations, delusions and sleep behavior); “affective” (depression and anxiety) and “apathy” (apathy/indifference and appetite/eating behavior).

### Statistical analysis

A descriptive analysis was performed according to the variable characteristics. Qualitative variables are expressed as frequencies and percentages while quantitative variables are expressed as means and standard deviations or median and interquartile range in skewed distributions.

The prevalence of caregiver burden (score ≥ 10 in the short ZBI in dementia), and the mean ZBI score, were estimated with their 95% confidence intervals (CIs). The normality of the Zarit test was verified using the Kolmogorov‒Smirnov test. The caregivers’ distress (global and caused by each NPS) was analysed using the NPI-D, studying it at each stage of dementia according to the GDS scale with its 95% CIs.

The association of burden according to the short ZBI with the sociodemographic variables of both patient and caregiver and patient clinical variables, including the presence or absence of each NPS and the total NPI score, was analysed using the chi-square test for qualitative variables and Student’s t test for quantitative variables. The association of the short ZBI score with the total NPI score and with the intensity of symptoms and subsyndromes was analysed using the Spearman correlation coefficient, and its association with the number of symptoms was analysed using ANOVA. The analysis was performed for each NPS separately, for NPSs grouped into subsyndromes, and as a whole (total NPI score).

To explain the factors associated with caregiver burden, multivariate models were constructed that considered the burden measured with the short ZBI (value ≥ 10) as the dependent variable and the caregiver and patient sociodemographic variables, patient clinical variables and the NPSs with statistical significance in the bivariate analysis and/or with clinical relevance as the independent variables. In model construction, the NPSs were each analysed according to their frequency and intensity, grouped into subsyndromes and as a whole (NPI scale).

Statistical analyses were performed with SPSS version 26 and STATA 14.

### Ethics approval

This study was conducted following the principles of the Declaration of Helsinki and its subsequent revisions and was approved by the Clinical Research Ethics Committee of Alcorcón Foundation University Hospital on 23 September 2015.

## Results

Of the 176 patients with dementia who met the inclusion criteria, 154 patients and caregivers could be located. A total of 125 caregivers of 129 patients agreed to participate. Four caregivers each cared for two patients. The study flowchart can be found in García-Martín et al. 2022 [28].

The mean age of the caregivers was 60.7 (± 14.3) and 77.6% were women. Among them, 79.2% had a secondary level of education or higher and 36.8% were employed. With respect to kinship, 47.2% of the caregivers were children of the patients, 33.6% were spouses and 10.4% were professional caregivers. A total of 80.8% lived with the patient, and 89.9% provided care 6–7 days a week. The sociodemographic characteristics of the caregivers and the sociodemographic and clinical characteristics of the patients who participated in the study are shown in Table 1 and **Supplement 1**.

**Table 1:** Caregivers’ characteristics and their relationship with burden

	n = 125		ZBI burden (≥ 10 points)	No ZBI burden (< 10 points)	p
	n (%)		% (95% CI)	% (95% CI)	
Age					
< 60 years	55 (44)		83.6 (71.2;92.2)	16.4 (7.8;28.8)	0.207
≥ 60 years	70 (56)		74.3 (62.4;84.0)	25.7 (16.0;37.6)	
Gender					
Male	28 (22.4)		67.9 (47.6;84.1)	32.1 (15.9;52.4)	0.124
Female	97 (77.6)		81.4 (72.3;88.6)	18.6 (11.4;27.7)	
Educational level					
Less than 5 years of education or primary education	26 (20.8)		80.8 (60.6;93.4)	19.2 (6.6;39.4)	0.741
Secondary, Bachelor’s or higher	99 (79.2)		77,8 (68.3;85.5)	22.2 (14.5;31.7)	
Employment status					
Employed	46 (36.8)		84.8 (71.1;93.7)	15.2 (6.3;28.9)	0.464
Homemaker	23 (18.4)		78.3 (56.3;92.5)	21.7 (7.5;43.7)	
Unemployed or retired	51 (40.8)		74.5 (60.4;85.7)	25.5 (14.3;39.6)	
Student or other	5 (4.0)		60.0 (14,7;94.7)	40.0 (5.3;85.3)	
Marital status					
Married	91 (72.8)		76.9 (66.9;85.1)	23.1 (14.9;33.1)	0.696
Single	19 (15.2)		78.9 (54.4;93.9)	21.1 (6.1;45.6)	
Separated, divorced or widower	15 (12.0)		86.7 (59.5;98.3)	13.3 (1.7;40.5)	
Kinship to the patient					
Spouse	42 (33.6)		78.6 (63.2;89.7)	21.4 (10.3;36.8)	0.963
Child	59 (47.2)		79.7 (67.2;89.0)	20.3 (11.0;32.8)	
Other relative	11 (8.8)		72.7 (39.0;94.0)	27.3 (6.0;61.0)	
Professional caregiver	13 (10.4)		76.9 (46.2;95.0)	23.1 (5.0;53.8)	
Lives with the patient*					
Yes	101 (80.8)		82.2 (73.3;89.1)	17.8 (10.9;26.7)	**0.035**
No	24 (19,2)		62.5 (40.6;81.2)	37.5 (18.8;59.4)	
Time caring for patient (n = 129)					
≤ 1 year	15 (11.6)		66.7 (38.4;88.2)	33.3 (11.8;61.6)	0.329
> 1 year − 10 years	99 (76.8)		79.8 (70.5;87.2)	20.2 (12.8;29.5)	
> 10 years	15 (11.6)		66.7 (38.4;88.2)	33.3 (11.8;61.6)	
Number of days per week of care (n = 129)					
1–3 days	6 (4.7)		50.0 (11.8;88.2)	50.0 (11.8;88.2)	0.251
4–5 days	7 (5.4)		85.7 (42.1;99.6)	14.3 (0.4;57.9)	
6–7 days	116 (89.9)		77.6 (68.9;84.8)	22.4 (15.2;31.1)	
Number of hours per day of care (n = 129) *					
≤ 8 h	24 (18.6)		54.2 (32.8;74.4)	45.8 (25.6;67.2)	**0.009**
> 8 − 12 h	13 (10.1)		92.3 (64.0;99.8)	7.7 (0.2;36.0)	
> 12 h	92 (71.3)		80.4 (70.9;88.0)	19.6 (12.0;29.1)	

* statistically significant

### Caregiver burden according to the short ZBI in dementia

A total of 78.4% (95% CI 71.0; 86.0) of the caregivers experienced burden according to the short ZBI in dementia. The burden was greater when the caregiver lived in the same home as the patient (82.2% vs. 62.5%) (p = 0.035) or if he or she provided more than 8 h of care per day ( p = 0.009). Greater burden was also found among younger caregivers (83.6% in those under 60 years of age), in women (81.4%) and in caregivers who worked outside the home (84.8%) (Table 1**).**

In terms of patient characteristics, caregiver burden was significantly related (p = 0.002) to low educational level in patients but not to their gender, age or level of independence. Caregivers presented greater burden if the patient had moderate dementia (stage GDS5) (83.3%) compared with those in milder or more severe stages. They also experienced greater burden if the patient lived with the family (81.1%) than when he or she lived alone (50.0%) or with a professional caregiver (60.0%) (p = 0.035) (**Supplement 1**).

### Relationship between caregiver burden and NPSs

The mean ZBI score of the caregivers was 12.3 (95% CI 11.6; 12.9). The score progressively increased according to the number of NPSs in the patient and ranged from 6.5 (95% CI -12.6; 25.6) in caregivers who cared for patients without NPSs to 13.6 (95% CI 8.4; 18.8) in caregivers caring for patients with 10 NPSs (p = 0.042) (**Supplement 2**).

**Fig. 1:**
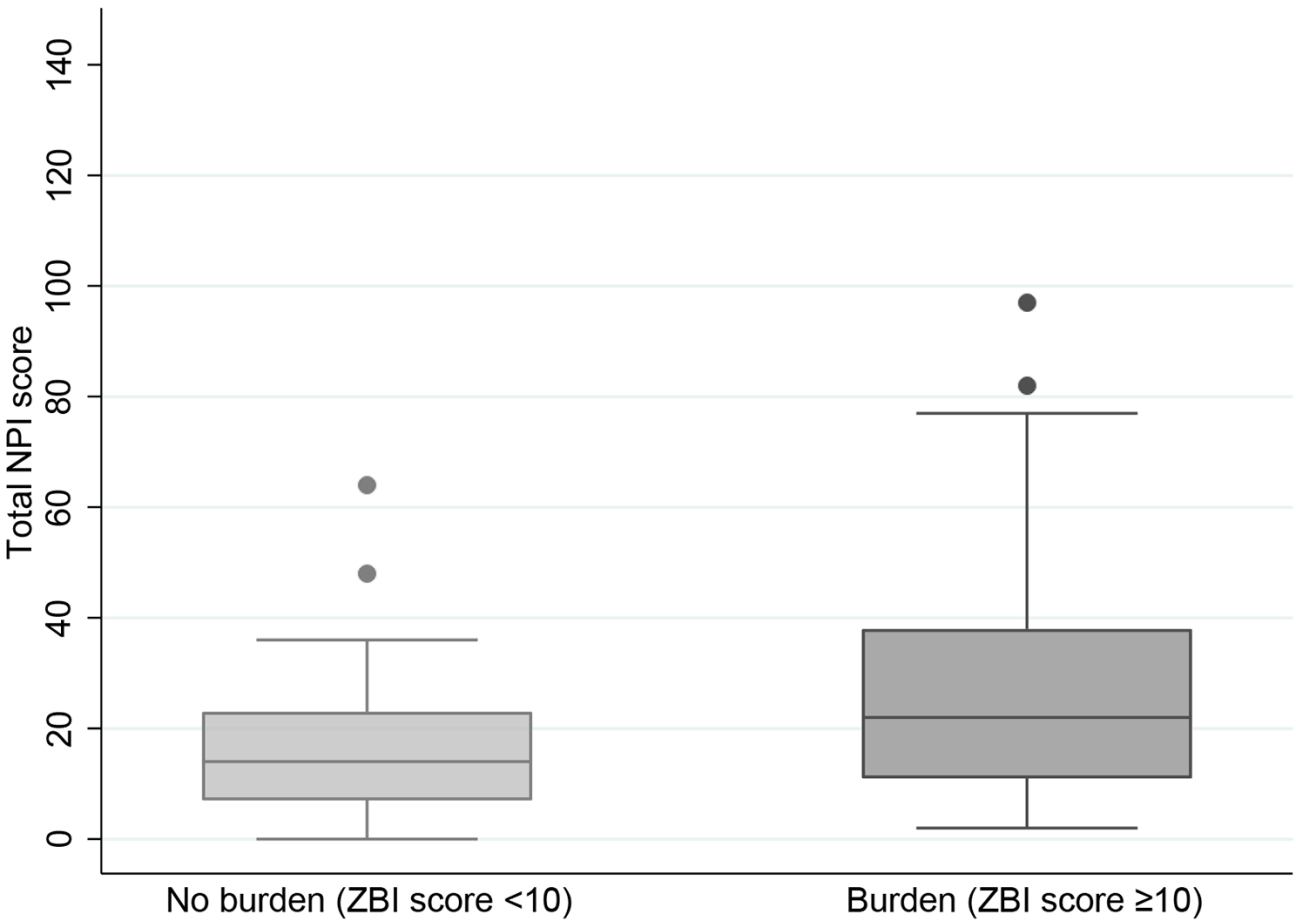
Caregiver burden according to patient total NPI score

The mean score on the NPI, which measures the intensity of patient symptoms, was 24.9 (95% CI 21.5; 28.4) with a median of 21.0 (IQR: 10.8–34.0). Caregiver burden was associated with a higher patient NPI score: the mean NPI score was 27.3 (95% CI 23.2; 31.5) and the median was 22.0 (IQR: 11.5–38.0) in patients whose caregivers experienced burden and 17.0 (95% CI 11.6; 22.5) and median of 14.0 (IQR: 7.0–23.0) in patients whose caregivers who did not experience burden (p = 0.003 and p = 0.083 respectively) (Fig. 1).

The NPSs whose presence was significantly associated with caregiver burden according to the ZBI score were disinhibition (93.5%), irritability (87.3%) and agitation (86.1%), while the subsyndrome most associated with burden was hyperactivity (82.9%) (Table 2).

**Table 2:** Relationship between caregiver burden and the presence of neuropsychiatric symptoms and subsyndromes

Neuropsychiatric symptoms	Burden (ZBI ≥ 10 points)		No burden (ZBI < 10 points)		p
	**n**	**% (95% CI)**	**n**	**% (95% CI)**	
Disinhibition*	43	93.5 (82.1;98.6)	3	6.5 (1.4;17.9)	**0.001**
Irritability/lability*	55	87.3 (76.5;94.4)	8	12.7 (5.6;23.5)	**0.006**
Agitation/aggression*	62	86.1 (75.9;93.1)	10	13.9 (6.9;24.1)	**0.005**
Aberrant motor behavior	35	87.5 (73.2;95.8)	5	12.5 (4.2;26.8)	0.053
Elation/euphoria	18	81.8 (59.7;94.8)	4	18.2 (5.2;40.3)	0.536
Apathy/indifference	70	77.8 (67.8;85.9)	20	22.2 (14.1;32.2)	0.673
Appetite/eating behavior	32	82.1 (66.5;92.5)	7	17.9 (7.5;33.4)	0.348
Delusions	40	76.9 (63.2;87.5)	12	23.1 (12.5;36.8)	0.968
Hallucinations	38	77.6 (63.4;88.2)	11	22.4 (11.8;36.6)	0.968
Sleep behavior	47	75.8 (63.3;85.8)	15	24.2 (14.2;36.7)	0.808
Depression/dysphoria	46	75.4 (62.7;85.5)	15	24.6 (14.5;37.3)	0.734
Anxiety	47	83.9 (71.7;92.4)	9	16.1 (7.6;28.3)	0.091
**Neuropsychiatric subsyndromes**					
Hyperactivity *	92	82.9 (74.6;89.4)	19	17.1 (10.6;25.4)	**0.000**
Apathy	79	79.0 (69.7;86.5)	21	21.0 (13.5;30.3)	0.260
Psychosis	68	79.1 (68.9;87.1)	18	20.9 (12.9;31.0)	0.377
Affective	66	78.6 (68.3;86.8)	18	21.4 (13.2;31.7)	0.502

* statistically significant

A positive correlation was found between the ZBI score and symptom intensity (frequency x severity) as measured by the NPI (Spearman’s correlation coefficient of 0.34, p < 0.001). The association was statistically significant for symptoms of anxiety, apathy, disinhibition, irritability and aberrant motor activity and the subsyndromes of hyperactivity, apathy and psychosis (Table 3).

**Table 3:** Correlation between the ZBI score and the intensity of each neuropsychiatric symptom or subsyndrome

Neuropsychiatric symptoms and subsyndromes	r	p
**Symptom intensity (frequency x severity)**		
Disinhibition*	0.334	**0.000**
Irritability/lability*	0.243	**0.006**
Agitation/aggression	0.167	0.058
Aberrant motor behavior*	0.187	**0.034**
Elation/euphoria	-0.018	0.838
Apathy/indifference*	0.198	**0.025**
Appetite/eating behavior	0.121	0.171
Delusions	0.173	0.050
Hallucinations	0.084	0.346
Sleep behavior	0.059	0.504
Depression/dysphoria	0.102	0.248
Anxiety*	0.217	**0.014**
**Subsyndrome intensity**		
Hyperactivity*	0.331	**0.000**
Apathy*	0.228	**0.009**
Psychosis*	0.197	**0.025**
Affective	0.159	0.073

r: correlation coefficient, *statistically significant

In the multivariate analysis, caregiver burden increased with each point on the NPI (OR 1.0, 95% CI 1.0; 1.1). Other factors associated with burden were female gender (OR 6.0, 95% CI 1.6; 22.8), working outside the home (OR 7.6, 95% CI 1.8; 31.8), spending ≥ 8 h a day caring for the patient (OR 5.6, 95% CI 1.4; 22.8), being related to the patient (OR 5.4, 95% CI 1.0; 28.2), living with the patient (OR 4.5, 95% CI 1.1; 19.6) and a low level of education (OR 8.3, 95% CI 2.3; 30.3).

In Model 2, which analysed the NPSs grouped into subsyndromes, caregiver burden was associated with the intensity of the hyperactivity subsyndrome (OR 1.1, 95% CI 1.0; 1.2). In Model 3, where the NPSs were analysed separately, an association was found with the intensity of disinhibition (OR 2.6, 95% CI 1.1; 5.8) and irritability (OR 1.2, 95% CI 1.0; 1.6) (Table 4 shows the ORs for the rest of the sociodemographic variables for models 2 and 3).

**Table 4:** Factors associated with caregiver burden (short ZBI ≥ 10)

	Model 1^1^			Model 2^2^			Model 3^3^		
	OR	(95% CI)	p	OR	(95% CI)	p	OR	(95% CI)	p
Caregiver gender (male)	6.0	(1.6; 22.8)	0.008	5.7	(1.4; 23.1)	0.014	6.0	(1.4; 26.1)	0.016
Employment status (Does not work)	7.6	(1.8; 31.8)	0.006	7.4	(1.7; 31.9)	0.008	7.7	(1.5; 39.4)	0.014
Kinship (No)	5.4	(1.0; 28.2)	0.046	5.3	(1.0; 28.8)	0.056	4.4	(0.7; 27.3)	0.116
Lives with patient (No)	4.5	(1.1; 19.6)	0.042	4.2	(0.9; 19.1)	0.065	6.0	(1.2; 30.1)	0.030
h of care (< 8 h)	5.6	(1.4; 22.8)	0.017	5.6	(1.3; 23.8)	0.021	5.1	(1.1; 23.7)	0.038
Patient education (≥ secondary)	8.3	(2.3; 30.3)	0.001	9.0	(2.4; 34.1)	0.001	12.3	(2.9; 53.3)	0.001
Total NPI^1^	1.0	(1.0; 1.1)	0.051						
Hyperactivity subsyndrome^2^				1.1	(1.0; 1.2)	0.003			
Disinhibition intensity^3^							2.6	(1.1; 5.8)	0.023
Irritability intensity^3^							1.2	(1.0; 1.6)	0.069

^1^ logistic regression model adjusted for NPI score (-2LL 100,578)

^2^ logistic regression model adjusted for the intensity of neuropsychiatric subsyndromes (-2LL 91,848)

^3^ logistic regression model adjusted for the intensity of neuropsychiatric symptoms (-2LL 83,755)

When analysing the NPSs according to their frequency instead of intensity, the NPSs most associated with caregiver burden were the presence of disinhibition with an OR of 12.7 (95% CI 2.5; 65.1) (p = 0.002), aberrant motor activity with an OR of 7.5 (95% CI 1.6; 33.8) (p = 0.009) and irritability with an OR of 3.0 (95% CI 0.9; 9.7) (p = 0.071) (**Supplement 3**).

### Caregiver distress according to the NPI-D score for NPSs

**Fig. 2:**
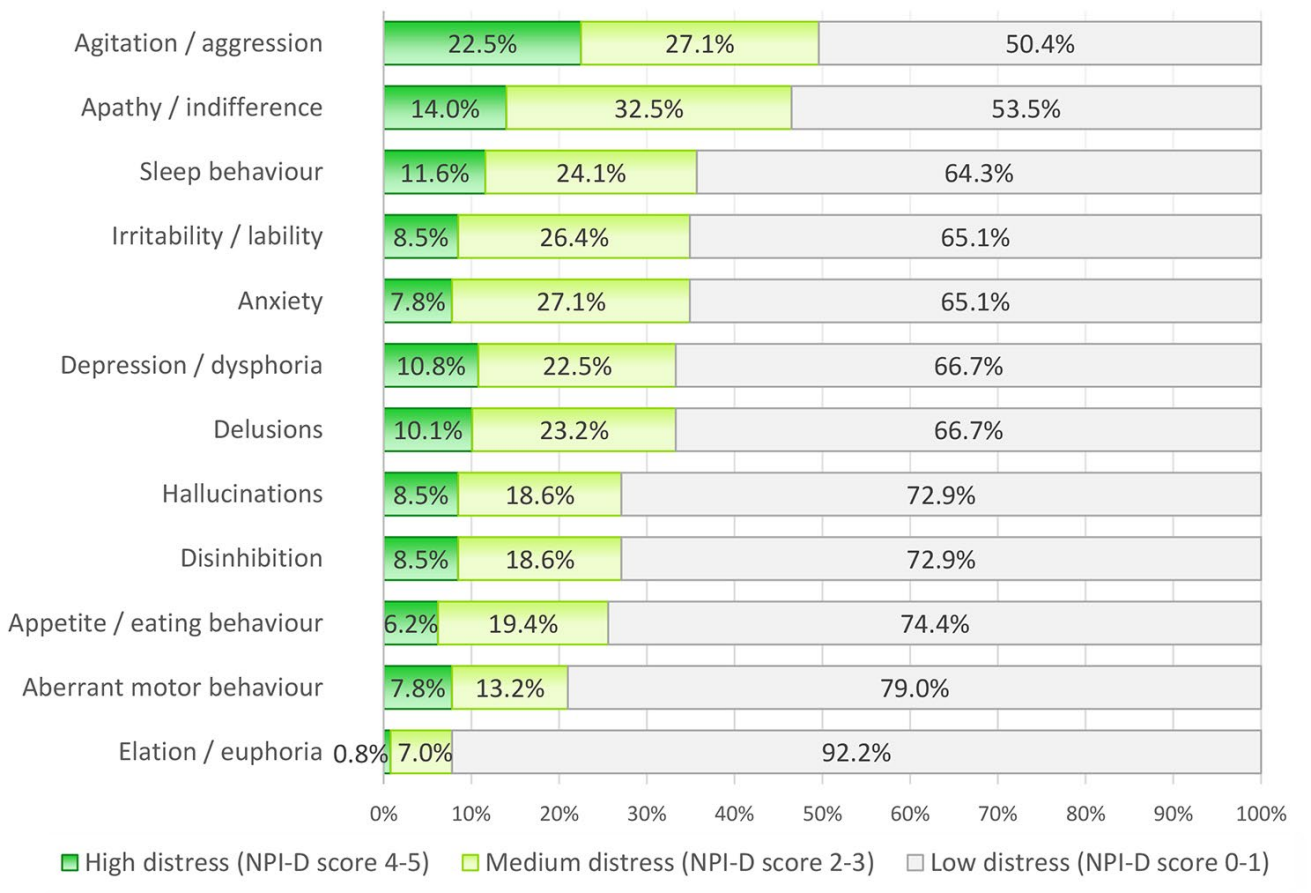
Caregiver distress according to neuropsychiatric symptoms (NPI-D score) classified according to the Kaufer model [25]

The mean caregiver distress score measured by the NPI-D was 12.5 (95% CI 11.1; 13.9). The symptoms that caused the greatest distress according to this scale were agitation, apathy and sleep disorders (Fig. 2 and **Supplement 4)**.

**Fig. 3:**
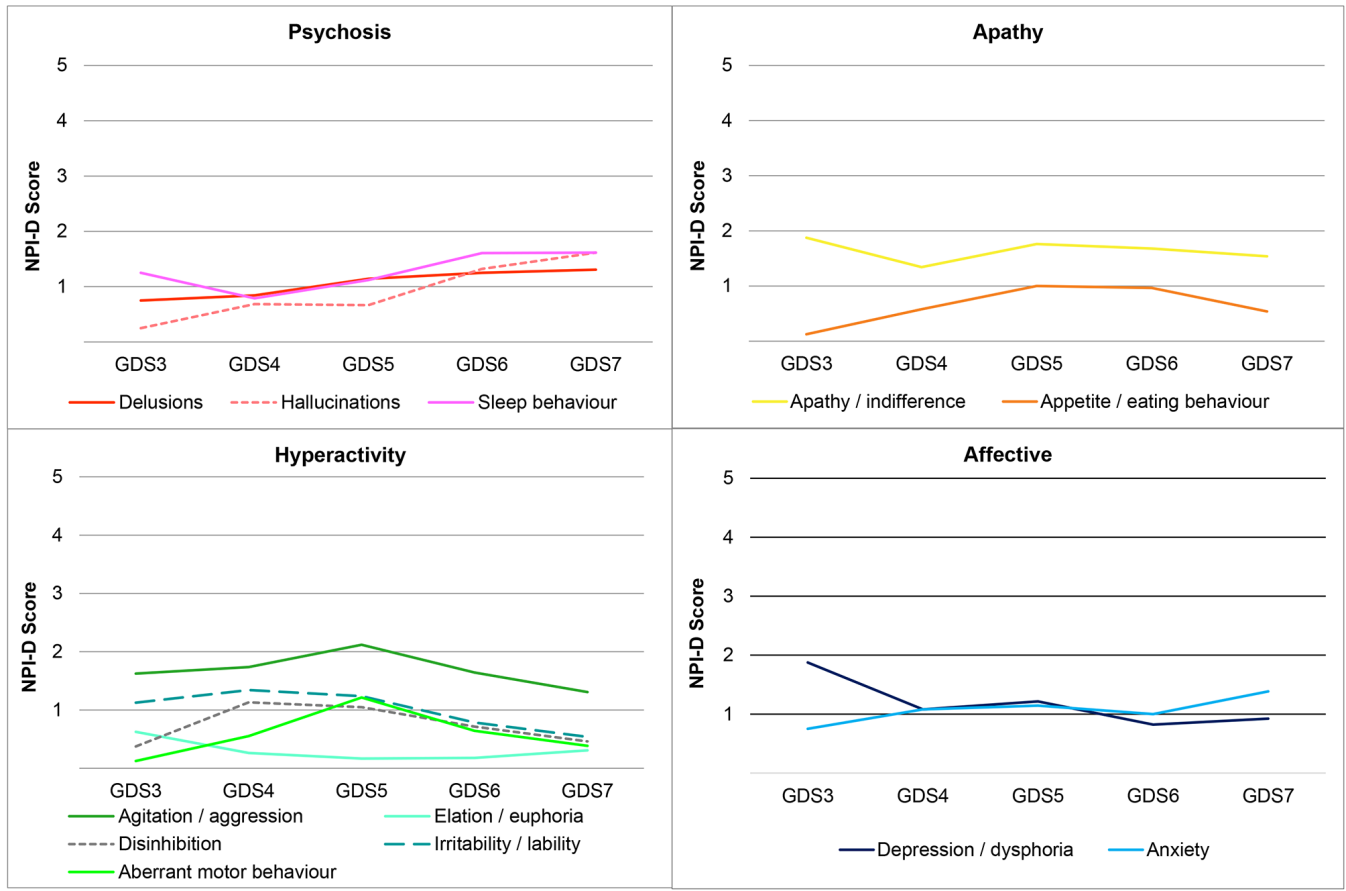
Caregiver distress related to neuropsychiatric symptoms (NPI-D score) grouped into subsyndromes according to the stage of dementia (GDS) GDS: Global Deterioration Scale

When analysing caregiver distress according to patient dementia stage, it was observed that depression caused more distress in mild dementia (stage GDS3) with an NPI-D score of 1.9 (95% CI 0.4; 3.3). Irritability, disinhibition, agitation and aberrant motor activity, all of which are symptoms of hyperactivity, caused greater distress in cases of mild (GDS4) and moderate (GDS5) dementia, with NPI-D scores between 0.6 (95% CI 0.2; 0.9) and 2.1 (95% CI 1.5; 2.7), while psychosis symptoms (delusions, hallucinations and sleep disorders) caused greater distress in advanced stages (NPI-D score between 1.3 (95% CI 0.7; 1.8) and 1.6 (95% CI 0.9; 2.3) in stages GDS 6 and 7). The distress produced by apathy remained more stable throughout all phases of dementia, with the NPI-D score varying between 1.3 (95% CI 0.9; 1.8) and 1.9 (95% CI 0.4; 3.3) (see Fig. 3 and **Supplement 5**).

## Discussion

The burden in caregivers of patients with dementia is high and is associated with the number of hours of care provided and the presence of NPSs. The NPSs that cause the most burden on caregivers are those grouped within the hyperactivity subsyndrome (agitation, irritability, disinhibition, aberrant motor activity), but the symptoms that cause greater distress to caregivers vary depending on the severity of dementia.

### Caregiver burden according to the short ZBI

In our study, almost 80% of all caregivers presented burden according to the short ZBI in dementia, and the burden was greater among those who lived with the patient, provided more than 8 h of care per day and worked outside the home. Other studies have also described the relationship between burden and patient–caregiver cohabitation [8, 38] and number of hours of care [12, 39, 40] but have reported no association with employment status [12, 38, 39, 41]. Three out of four caregivers were women, who presented greater burden, as reported in previous studies [8, 11, 21, 41, 42]. Burden was also higher among younger caregivers, although the difference in burden between younger and older caregivers was not significant.

In terms of patient characteristics, caregiver burden was associated with low educational level, a relationship that has already been described by previous studies [43, 44] but not with the age or gender of patients. The association between patient education and caregiver burden could be a result of patients with a lower educational level having a lower socioeconomic status, which may affect their access to resources and preclude sharing the burden of care, such as with day centers or professional caregivers, since studies show an inverse relationship between family or patient income and caregiver burden [38, 43, 44]. The severity of dementia has also been linked to caregiver burden [38, 41]. In our study, the time since onset and severity of the disease showed a similar pattern, with greater caregiver burden observed at intermediate stages (moderate dementia) than at initial or advanced stages (mild or severe dementia). No differences in burden were observed between caregivers of patients who were treated or not treated with specific drugs for dementia or between caregivers of patients who were treated or not treated with neuroleptics, antidepressants or benzodiazepines for NSPs.

When analysing the relationship between caregiver burden and NPSs, it was observed that both the presence and intensity of such symptoms worsened the burden. Thus, the ZBI score increased with the number and intensity of patient symptoms (total NPI score), such that caregivers with burden cared for patients with higher NPI scores than those without burden, a relationship already observed in previous studies [38, 45–47].

Caregiver burden was associated with the presence of disinhibition and irritability, which are two NPSs included in the hyperactivity subsyndrome and that are not easily treatable with drugs [48]. Apathy, agitation, aberrant motor activity and anxiety were only significant in bivariate models, while psychosis symptoms (delusions and hallucinations) were only significant when their intensity was considered, and thus, they were grouped into subsyndromes and analysed together.

Other studies have observed a relationship between the ZBI score and NPSs, although the results are not uniform. The NPSs most frequently associated with caregiver burden are irritability [13, 39, 43, 45, 46, 49], agitation [13, 14, 45, 46, 49], delusions [13, 45, 46, 49] and sleep disorders [13, 14, 39, 46]. In contrast, apathy [13, 43, 46], hallucinations [45, 46, 49] and anxiety [13, 21] are less frequently associated with burden. The association between burden and disinhibition and aberrant motor activity reported in the present study appears in fewer previous studies [46]. Euphoria is not only the least frequent symptom but also the one that results in the least burden.

The ZBI and its different short versions are commonly used to measure caregiver burden, although their use differs among studies, such as in the selection of different cutoff points for burden (e.g., 40 points [12], 20 points [50], median [39]) or application of only item 22, which refers to the “overall felt caregiver burden” [41], to give just a few examples. Other studies use scales other than the ZBI, such as the Brief Symptom Inventory (BSI-18) [51], Perceived Social Stress Scale [20] and Burden Scale for Family Caregivers (BSFC-s) [52], to measure caregiver burden. Here, we used the short ZBI in dementia, a brief version with only four items that is adapted to measure burden in caregivers of patients with dementia [30]. This version has high sensitivity and specificity, and therefore, it may be more useful for daily clinical practice than longer versions. We analysed its behavior as a numerical variable and a cutoff point for burden presence/absence, defined as ≥ 10 and < 10 points, respectively, to compare our findings with those of other studies and to determine whether the test allowed us to appropriately capture the burden associated with NPSs.

In addition to different ways of measuring burden, the NPSs measured with the NPI may also be approached differently according to the literature. In this study, we used several analytical models to determine which could best reflect caregiver burden. We found that models that separately analyse the frequency or intensity of NPSs or symptoms grouped in subsyndromes are more effective than those that use the total NPI score. In addition, these models have greater clinical implications as for which symptoms should be targeted to reduce caregiver burden.

### Caregiver distress according to the NPI-D

The NPI-D specifically measures caregiver distress related to NPSs, unlike the ZBI, which measures the overall burden on the caregiver. The mean NPI-D score was 12.5 (± 8.2), which is similar to that reported in a study whose patients had similar characteristics to ours [53] and is higher than that of others that included mostly patients with mild dementia [25, 54–57] or studies that used the 10-symptom version of the NPI instead of the 12-symptom version [58]. Agitation, apathy and sleep disorders are the symptoms that cause the greatest distress in caregivers. Agitation typically causes the greatest distress, along with delusions, irritability, disinhibition and aberrant motor activity [25, 54–56, 58, 59]; however, apathy [60] and sleep disorders [61] do not typically cause high distress.

### Contributions and strengths of our study

This study allows us to compare the results of two types of measures of caregiver burden and distress in the same population. The discrepancy between the NPSs that individually produce greater distress among caregivers and those that are ultimately associated with burden is striking. Thus, the presence of agitation in patients, which is the symptom that results in the greatest caregiver distress (high distress in a quarter of caregivers), is not significant in the burden model, while disinhibition, which causes less distress (high distress in only 8% of caregivers), appears to be associated with burden in the final model. This disagreement indicates the need to expand the number of studies that compare the results of both scales.

Another contribution of our study is the analysis of distress caused by NPSs according to the severity of dementia, an aspect that few studies have addressed [57]. It was observed that in mild stages (GDS3), depression (affective symptoms) caused more distress. In contrast, in mild and moderate dementia (GDS4 and 5), agitation, irritability and aberrant motor activity (hyperactivity symptoms), and in advanced dementia (GDS6 and 7), delusions, hallucinations and sleep disturbances (psychosis symptoms), caused the most distress. This difference is probably related to the variations in frequency and intensity of the different NPSs as dementia progresses [28] and can explain the variability in caregiver burden reported in different studies, which also depends on the characteristics of the patients included. This difference could also explain why we did not find a clear association between psychosis symptoms and burden, as these symptoms occur in advanced stages of dementia, which had less representation in our study. Using a different approach, a Korean study also found an indirect relationship between dementia stages and burden on caregivers of patients who presented with psychosis symptoms and physical behavior symptoms (apathy, aberrant motor activity, sleep and appetite disorders) [57].

There are few studies carried out in primary care where the burden of caregivers of patients with dementia and its relationship with neuropsychiatric symptoms (NPS) has been analysed. There are also few studies on this subject carried out in Spain [23, 62] Therefore, our study can provide knowledge in this area, highlighting the relationship between the presence of NPSs in patients and the burden on caregivers.

### Limitations

With regard to the limitations of our study, the use of the short version of the ZBI in dementia does not allow us to compare the mean values obtained with studies that used the 22-item version [12, 39–41, 43, 45, 50, 58, 62–67] or valued obtained with short versions different from ours [38, 47, 68, 69]. However, the behavior of the short ZBI with respect to the NPSs is similar to that found with other short versions and with the original version. Taking this similarity into account, the short ZBI in dementia may be a good alternative for use in patients with cognitive decline, as it is quicker to apply and provides similar results.

Factors that can alleviate the burden on caregivers, such as social benefits, were not analysed due to insufficient data in all patients. At least 28 of our patients received external help, but other patients may also have been receiving unreported help (day centers or mixed caregivers). The interviews were conducted with a single caregiver per patient, but whether care was shared with another caregiver was not considered. We found that many patients had mixed caregivers (several relatives or close family and professional caregivers), who sometimes shared the responsibility of care in a very homogeneous way. In Spain, the National Health System is universal and free. Social aids such as residences, day centers, home aids or technical aids are not systematically implemented but must be requested. After evaluation by specialized teams, they may be implemented free or with shared payment according to the income level of the patients and their family support network [70].

### Future lines of research

It is known that caregiver burden causes early patient institutionalization [42]. Analysing the modifiable factors that contribute to this burden can help control it, thus improving the quality of life of caregivers and delaying institutionalization. It is important to continue research in this field, including a vision of gender perspective, global workloads inside and outside the home, level of education, own resources and/or access to different types of help and beliefs about caring for relatives. The latter aspect can mark important differences between the populations of different regions or countries.

Additionally, as we have commented, we find it interesting to further investigate the NPSs in the stages of dementia and compare different scales to see which may be the most appropriate to detect the burden of caregivers in PC and be able to act on it as soon as possible.

Finally, research in PC, where patients and caregivers receive care, seems essential to us so that the results have greater applicability.

## Conclusions

This study relates the burden experienced by caregivers with their workload and with patients’ neuropsychiatric symptoms and describes the symptoms that cause caregivers greater distress according to each dementia stage.

Based on the results, reducing the burden on caregivers by decreasing the hours of care provided through external help or by controlling NPSs could relieve caregiver burden and its consequences on patients. Knowing which NPSs are more frequent in each stage of dementia and when they cause more distress could allow the implementation of specific support measures aimed at reducing burden adapted to each stage of the disease. Caregiver training is a fundamental pillar within these measures, especially in the management of hyperactivity symptoms, such as aberrant motor activity and disinhibition, which do not have an easy pharmacological solution and for which it is advisable to prioritize nonpharmacological management.

### Electronic supplementary material

Below is the link to the electronic supplementary material.

Supplementary Material 1

Supplementary Material 2

Supplementary Material 3

Supplementary Material 4

Supplementary Material 5

## Data Availability

The datasets used and analysed during the current study are available from the corresponding author (vgarcia@salud.madrid.org) on reasonable request. All data generated or analysed during this study are included in this published article and its supplementary information files.

## List of abbreviations

BPSD: Behavioral and psychological symptoms of dementia
CD: Cognitive decline
ChEI: Cholinesterase inhibitor
EHRs: Electronic Health Records
GDS: Global Deterioration Scale
ICPC: International Classification of Primary Care
NPI: Neuropsychiatric Inventory
NPI-D: Neuropsychiatric Inventory Caregiver Distress Scale
NPSs: Neuropsychiatric symptoms
STROBE: STrengthening the Reporting of OBservational studies in Epidemiology
ZBI: Zarit Burden Interview

## References

[1] Cao Q, Tan CC, Xu W, Hu H, Cao XP, Dong Q (2020). The prevalence of dementia: a systematic review and Meta-analysis. J Alzheimers Dis.

[2] Nichols E, Steinmetz JD, Vollset SE, Fukutaki K, Chalek J, Abd-Allah F (2022). Estimation of the global prevalence of dementia in 2019 and forecasted prevalence in 2050: an analysis for the global burden of Disease Study 2019. Lancet Public Health.

[3] Sontheimer N, Konnopka A, König HH (2021). The excess costs of dementia: a systematic review and Meta-analysis. J Alzheimer’s Disease.

[4] Marešová P, Dolejs J, Mohelska H, Bryan LK (2020). Cost of treatment and care for people with Alzheimer’s Disease: a Meta- analysis. Curr Alzheimer Res.

[5] Takizawa C, Thompson PL, van Walsem A, Faure C, Maier WC (2015). Epidemiological and economic burden of Alzheimer’s disease: a systematic literature review of data across Europe and the United States of America. J Alzheimer’s Disease.

[6] Cantarero-Prieto D, Leon PL, Blazquez-Fernandez C, Juan PS, Cobo CS (2020). The economic cost of dementia: a systematic review. Dement (London).

[7] Cepoiu-Martin M, Tam-Tham H, Patten S, Maxwell CJ, Hogan DB (2016). Predictors of long-term care placement in persons with dementia: a systematic review and meta-analysis. Int J Geriatr Psychiatry.

[8] Schaffler-Schaden D, Krutter S, Seymer A, Eßl-Maurer R, Flamm M, Osterbrink J (2021). Caring for a relative with dementia: determinants and gender differences of Caregiver Burden in the rural setting. Brain Sci.

[9] Gresswell I, Lally L, Adamis D, McCarthy GM (2018). Widening the net: exploring social determinants of burden of informal carers. Ir J Psychol Med.

[10] Swinkels J, van Tilburg T, Verbakel E, Broese van Groenou M (2017). Explaining the gender gap in the Caregiving Burden of Partner Caregivers. Journals of Gerontology Series B: Psychological Sciences and Social Sciences.

[11] Xiong C, Biscardi M, Astell A, Nalder E, Cameron JI, Mihailidis A (2020). Sex and gender differences in caregiving burden experienced by family caregivers of persons with dementia: a systematic review. McMunn A, editor. PLoS ONE.

[12] Lucijanić J, Baždarić K, Librenjak D, Lucijanić M, Hanževački M, Jureša V (2020). A validation of the croatian version of Zarit Burden interview and clinical predictors of caregiver burden in informal caregivers of patients with dementia: a cross-sectional study. Croat Med J.

[13] Terum TM, Andersen JR, Rongve A, Aarsland D, Svendsboe EJ, Testad I (2017). The relationship of specific items on the neuropsychiatric inventory to caregiver burden in dementia: a systematic review. Int J Geriatr Psychiatry.

[14] Ornstein K, Gaugler JE (2012). The problem with “problem behaviors”: a systematic review of the association between individual patient behavioral and psychological symptoms and caregiver depression and burden within the dementia patient-caregiver dyad. Int Psychogeriatr.

[15] Zhao QF, Tan L, Wang HF, Jiang T, Tan MS, Tan L (2016). The prevalence of neuropsychiatric symptoms in Alzheimer’s disease: systematic review and meta-analysis. J Affect Disord.

[16] Peters ME, Schwartz S, Han D, Rabins PV, Steinberg M, Tschanz JT (2015). Neuropsychiatric symptoms as predictors of progression to severe Alzheimer’s dementia and death: the cache county dementia progression study. Am J Psychiatry.

[17] Risco E, Cabrera E, Jolley D, Stephan A, Karlsson S, Verbeek H (2015). The association between physical dependency and the presence of neuropsychiatric symptoms, with the admission of people with dementia to a long-term care institution: a prospective observational cohort study. Int J Nurs Stud.

[18] Afram B, Stephan A, Verbeek H, Bleijlevens MHC, Suhonen R, Sutcliffe C (2014). Reasons for institutionalization of people with dementia: Informal Caregiver Reports from 8 european countries. J Am Med Dir Assoc.

[19] Tun SM, Murman DL, Long HL, Colenda CC, Von Eye A (2007). Predictive validity of neuropsychiatric subgroups on nursing home placement and survival in patients with alzheimer disease. Am J Geriatric Psychiatry.

[20] Flores D, Rote S, Angel J, Chen NW, Downer B, Markides K (2021). Depressive symptoms in child caregivers of very old Mexican Americans. Aging Ment Health.

[21] Regier NG, Hodgson NA, Gitlin LN (2020). Neuropsychiatric symptom profiles of community-dwelling persons living with dementia: factor structures revisited. Int J Geriatr Psychiatry.

[22] Michelet M, Lund A, Strand BH, Engedal K, Selbaek G, Bergh S (2020). Characteristics of patients assessed for cognitive decline in primary healthcare, compared to patients assessed in specialist healthcare. Scand J Prim Health Care.

[23] Viñas Díez V, Conde Sala JL, Turró Garriga O, Gascón Bayarri J, Reñé Ramirez R (2019). Síntomas depresivos y sobrecarga en los familiares cuidadores en la enfermedad de Alzheimer: un modelo de ecuaciones estructurales. Rev Neurol.

[24] Tu JY, Jin G, Chen JH, Chen YC (2022). Caregiver Burden and Dementia: a systematic review of Self-Report Instruments. J Alzheimer’s Disease.

[25] Kaufer DI, Cummings JL, Christine D, Bray T, Castellon S, Masterman D (1998). Assessing the impact of neuropsychiatric symptoms in Alzheimer’s disease: the neuropsychiatric inventory caregiver distress scale. J Am Geriatr Soc.

[26] Vilalta-Franch J, Lozano-Gallego M, Hernández-Ferrándiz M, Llinàs-Reglà J, López-Pousa SLO (1999). Neuropsychiatric inventory: propiedades psicométricas de su adaptación al español. Rev Neurol.

[27] Boada M, Cejudo JC, Tàrraga L, López OL, Kaufer D (2002). Neuropsychiatric inventory questionnaire (NPI-Q): spanish validation of an abridged form of the neuropsychiatric inventory (NPI). Neurologia.

[28] García-Martín V, de Hoyos-Alonso MC, Ariza-Cardiel G, Delgado-Puebla R, García-Domingo P, Hernández-Melo E (2022). Neuropsychiatric symptoms and subsyndromes in patients with different stages of dementia in primary care follow-up (NeDEM project): a cross-sectional study. BMC Geriatr.

[29] Vandenbroucke JP, von Elm E, Altman DG, Gøtzsche PC, Mulrow CD, Pocock SJ (2007). Strengthening the reporting of Observational Studies in Epidemiology (STROBE): explanation and elaboration. PLoS Med.

[30] Gort A, Mingot M, March J, Gómez X, Soler T, Nicolás F (2010). Utilidad de la escala de Zarit reducida en demencias. Med Clin (Barc).

[31] Zarit SH, Reever KE, Bach-Peterson J (1980). Relatives of the impaired Elderly: correlates of feelings of Burden. Gerontologist.

[32] Martín M, Salvadó I, Nadal S, Miji L, Rico J. Adaptación para nuestro medio de la escala de sobrecarga del cuidador (Caregiver Burden interview) de Zarit. Revista de Gerontología. 1996;338–45.

[33] Mahoney FI, Barthel DW (1965). Functional evaluation: the Barthel Index. Md State Med J.

[34] Shah S, Vanclay F, Cooper B (1989). Improving the sensitivity of the Barthel Index for stroke rehabilitation. J Clin Epidemiol.

[35] Reisberg B, Ferris SH, De Leon MJ, Crook T (1982). The global deterioration scale for assessment of primary degenerative dementia. Am J Psychiatry.

[36] Cummings JL (1997). The neuropsychiatric inventory: assessing psychopathology in dementia patients. Neurology.

[37] Aalten P, Verhey FRJ, Boziki M, Bullock R, Byrne EJ, Camus V (2007). Neuropsychiatric Syndromes in Dementia: results from the european Alzheimer Disease Consortium - Part I. Dement Geriatr Cogn Disord.

[38] Ku LJE, Chang SM, Pai MC, Hsieh HM (2019). Predictors of caregiver burden and care costs for older persons with dementia in Taiwan. Int Psychogeriatr.

[39] Sutcliffe C, Giebel C, Bleijlevens M, Lethin C, Stolt M, Saks K (2017). Caring for a person with dementia on the margins of long-term care: a perspective on Burden from 8 european countries. J Am Med Dir Assoc.

[40] Germain S, Adam S, Olivier C, Cash H, Ousset PJ, Andrieu S (2009). Does cognitive impairment influence burden in caregivers of patients with alzheimer’s disease?. J Alzheimer’s Disease.

[41] Ransmayr G, Hermann P, Sallinger K, Benke T, Seiler S, Dal-Bianco P (2018). Caregiving and caregiver burden in dementia home care: results from the prospective dementia registry (PRODEM) of the austrian Alzheimer Society. J Alzheimer’s Disease.

[42] Etters L, Goodall D, Harrison BE (2008). Caregiver burden among dementia patient caregivers: a review of the literature. J Am Acad Nurse Pract.

[43] Tsai CF, Hwang WS, Lee JJ, Wang WF, Huang LC, Huang LK et al. Predictors of caregiver burden in aged caregivers of demented older patients. BMC Geriatrics 2021 21:1. 2021;21(1):1–9. 10.1186/s12877-021-02007-1 PMC7809883 33446114

[44] Chiao CY, Wu HS, Hsiao CY (2015). Caregiver burden for informal caregivers of patients with dementia: a systematic review. Int Nurs Rev.

[45] Wong CSC, Zelman DC (2020). Caregiver expressed emotion as mediator of the relationship between neuropsychiatric symptoms of dementia patients and caregiver mental health in Hong Kong. Aging Ment Health.

[46] Baharudin AD, Din NC, Subramaniam P, Razali R. The associations between behavioral-psychological symptoms of dementia (BPSD) and coping strategy, burden of care and personality style among low-income caregivers of patients with dementia. BMC Public Health. 2019;19(Suppl 4). 10.1186/s12889-019-6868-0 PMC6565534 31196141

[47] Dauphinot V, Ravier A, Novais T, Delphin-Combe F, Mouchoux C, Krolak-Salmon P (2016). Risk factors of Caregiver Burden Evolution, for patients with subjective cognitive decline or Neurocognitive Disorders: a longitudinal analysis. J Am Med Dir Assoc.

[48] Keszycki RM, Fisher DW, Dong H (2019). The hyperactivity-impulsivity-irritiability-disinhibition-aggression-agitation domain in Alzheimer’s disease: current management and future directions. Front Pharmacol.

[49] van der Linde RM, Dening T, Matthews FE, Brayne C (2014). Grouping of behavioural and psychological symptoms of dementia. Int J Geriatr Psychiatry.

[50] Liu S, Jin Y, Shi Z, Huo YR, Guan Y, Liu M (2017). The effects of behavioral and psychological symptoms on caregiver burden in frontotemporal dementia, Lewy body dementia, and Alzheimer’s disease: clinical experience in China. Aging Ment Health.

[51] Teipel SJ, Thyrian JR, Hertel J, Eichler T, Wucherer D, Michalowsky B (2015). Neuropsychiatric symptoms in people screened positive for dementia in primary care. Int Psychogeriatr.

[52] Kürten L, Dietzel N, Kolominsky-Rabas PL, Graessel E. Predictors of the one-year-change in depressiveness in informal caregivers of community-dwelling people with dementia. BMC Psychiatry 2021 21:1. 2021;21(1):1–11. 10.1186/s12888-021-03164-8 PMC8019174 33812389

[53] De Vugt ME, Stevens F, Aalten P, Lousberg R, Jaspers N, Verhey FRJ (2005). A prospective study of the effects of behavioral symptoms on the institutionalization of patients with dementia. Int Psychogeriatr.

[54] Thyrian JR, Eichler T, Hertel J, Wucherer D, Dreier A, Michalowsky B (2015). Burden of behavioral and psychiatric symptoms in people screened positive for dementia in primary care: results of the DelpHi-study. J Alzheimer’s Disease.

[55] Lin PC, Lin HT, Yang YH, Yang YH (2020). The effects of caregiver characteristics on behavioral and psychological symptoms of dementia of patients with dementia. Aging Ment Health.

[56] Huang SS, Lee MC, Liao YC, Wang WF, Lai TJ (2012). Caregiver burden associated with behavioral and psychological symptoms of dementia (BPSD) in taiwanese elderly. Arch Gerontol Geriatr.

[57] Kim B, Noh GO, Kim K (2021). Behavioural and psychological symptoms of dementia in patients with Alzheimer’s disease and family caregiver burden: a path analysis. BMC Geriatr.

[58] Matsumoto N, Ikeda M, Fukuhara R, Shinagawa S, Ishikawa T, Mori T (2007). Caregiver burden associated with behavioral and psychological symptoms of dementia in elderly people in the local community. Dement Geriatr Cogn Disord.

[59] Balieiro AP, Sobreira EST, Pena MCS, Silva-Filho JH, do Vale FAC (2010). Caregiver distress associated with behavioral and psychological symptoms in mild alzheimer’s disease. Dement e Neuropsychologia.

[60] Rinaldi P, Spazzafumo L, Mastriforti R, Mattioli P, Marvardi M, Polidori MC (2005). Predictors of high level of burden and distress in caregivers of demented patients: results of an italian multicenter study. Int J Geriatr Psychiatry.

[61] Merrilees J, Hubbard E, Mastick J, Miller BL, Dowling GA (2014). Sleep in persons with frontotemporal dementia and their family caregivers. Nurs Res.

[62] Vilalta-Franch J, Ló Pez-Pousa S, Turon-Estrada A, Lozano-Gallego M (2010). Syndromic Association of behavioral and psychological symptoms of dementia in Alzheimer Disease and patient classification. J Geriatr Psychiatry.

[63] Gonfrier S, Andrieu S, Renaud D, Vellas B, Robert PH (2012). Course of neuropsychiatric symptoms during a 4-year follow up in the REAL-FR cohort. J Nutr Health Aging.

[64] Reed C, Belger M, Scott Andrews J, Tockhorn-Heidenreich A, Jones RW, Wimo A et al. Factors associated with long-term impact on informal caregivers during Alzheimer’s disease dementia progression: 36-month results from GERAS. Int Psychogeriatr. 2019;(2020):267–77. 10.1017/S1041610219000425 31134870

[65] Sheikh F, Ismail Z, Mortby ME, Barber P, Cieslak A, Fischer K (2018). Prevalence of mild behavioral impairment in mild cognitive impairment and subjective cognitive decline, and its association with caregiver burden. Int Psychogeriatr.

[66] Vaingankar JA, Chong SA, Abdin E, Picco L, Jeyagurunathan A, Seow E (2017). Behavioral and psychological symptoms of dementia: prevalence, symptom groups and their correlates in community-based older adults with dementia in Singapore. Int Psychogeriatr.

[67] Luchsinger JA, Tipiani D, Torres-Patiño G, Silver S, Eimicke JP, Ramirez M (2015). Characteristics and mental health of hispanic dementia caregivers in New York City. Am J Alzheimers Dis Other Demen.

[68] Cova I, Travi N, Maggiore L, Cucumo V, Mariani C, Pomati S (2018). What are the caregivers’ needs on dementia care? An integrated qualitative and quantitative assessment. Neurol Sci.

[69] Toda D, Tsukasaki K, Itatani T, Kyota K, Hino S, Kitamura T (2018). Predictors of potentially harmful behaviour by family caregivers towards patients treated for behavioural and psychological symptoms of dementia in Japan. Psychogeriatrics.

[70] Grupo de trabajo de la Guía de Práctica Clínica sobre la atención integral a las personas con enfermedad de Alzheimer y otras demencias. Guías de Práctica Clínica en el SNS: AIAQS Núm. 2009/07. Guía de Práctica Clínica sobre la atención integral a las personas con enfermedad de Alzheimer y otras demencias. Plan de Calidad para el Sistema Nacional de Salud del Ministerio de Sanidad, Política Social e Igualdad. Agència d’Informació, Avaluació i Qualitat en Salut de Cataluña; 2010.

