# Supplementary material for: Burden in caregivers of primary care patients with dementia: influence of neuropsychiatric symptoms according to disease stage (NeDEM project)
Source: BMC Geriatr. 2023 Aug 29;23:525. doi: 10.1186/s12877-023-04234-0 (PMC10463529; doi:10.1186/s12877-023-04234-0)
Supplement: Supplementary file 1 — Supplementary Material 1 [file 12877_2023_4234_MOESM1_ESM.docx]

**Supplement 1.** Patient characteristics and their relationship with caregiver burden.

| Patient characteristics (n = 129) | **n (%)** | **Caregiver burden according to the short ZBI for dementia (≥10 points)** | |
| --- | --- | --- | --- |
|  |  | **% (95% CI)** | **p** |
| Age |  |  |  |
| <65 years | 2 (1.6) | 50.0 (1.3;98.7) | 0.457 |
| 65-74 years | 21 (16.3) | 71.4 (47.8;88.7) |  |
| 75-79 years | 15 (11.6) | 66.7 (38.4;88.2) |  |
| ≥80 years | 91 (70.5) | 80.2 (70.6;87.8) |  |
| Gender |  |  |  |
| Male | 38 (29.5) | 81.6 (65.7;92.3) | 0.401 |
| Female | 91 (70.5) | 74.7 (64.5;83.2) |  |
| Educational level* |  |  |  |
| Illiterate, none or primary education | 103 (79.8) | 82.5 (73.8;89.3) | 0.002 |
| Secondary or university | 26 (20.2) | 53.8 (33.4;73.4) |  |
| GDS stage |  |  |  |
| GDS 3 (mild CD, borderline deterioration) | 8 (6.2) | 75.0 (34.9;96.8) | 0.594 |
| GDS 4 (moderate CD, mild dementia) | 38 (29.4) | 79.0 (62.7;90.4) |  |
| GDS 5 (moderately severe CD, moderate dementia) | 42 (32.6) | 83.3 (68.6;93.0) |  |
| GDS 6 (severe CD, moderately severe dementia) | 28 (21.7) | 67.9 (47.6;84.1) |  |
| GDS 7 (very severe CD, severe dementia) | 13 (10.1) | 69.2 (38.5;90.9) |  |
| Barthel Index |  |  |  |
| Independence (100 points) | 18 (14.0) | 72.2 (46.5;90.3) | 0.923 |
| Low dependence (91-99 points) | 6 (4.6) | 83.3 (35.9;99.6) |  |
| Moderate dependence (61-90 points) | 37 (28.7) | 78.4 (61.8;90.2) |  |
| Severe dependence (21-60 points) | 35 (27.1) | 80.0 (63.1;91.69 |  |
| Total dependence (<21 points) | 33 (25.6) | 72.7 (54.5;86.7) |  |
| Time since dementia onset |  |  |  |
| ≤1 year | 9 (7.0) | 77.8 (40.0;97.2) | 0.524 |
| >1-3 years | 39 (30.2) | 74.4 (57.9;87.0) |  |
| >3-6 years | 47 (36.5) | 83.0 (69.2;92.4) |  |
| >6-9 years | 15 (11.6) | 80.0 (51.9;95.7) |  |
| >9 years | 19 (14.7) | 63.2 (38.4;83.7) |  |
| Treatment for dementia |  |  |  |
| No specific treatment | 35 (27.1) | 74.3 (56.7;87.5) | 0.687 |
| Specific treatment: ChEIs and/or memantine | 94 (72.9) | 77.7 (67.9;85.6) |  |
| Neuroleptic treatment |  |  |  |
| No | 74 (57.4) | 74.3 (62.8;83.8) | 0.450 |
| Yes | 55 (42.6) | 80.0 (67.0;89.6) |  |
| Benzodiazepine treatment |  |  |  |
| No | 83 (64.3) | 75.9 (65.3;84.6) | 0.761 |
| Yes | 46 (35.7) | 78.3 (63.6;89.0) |  |
| Antidepressant treatment |  |  |  |
| No | 67 (51.9) | 74.6 (62.5;84.4) | 0.554 |
| Yes | 62 (48.1) | 79.0 (66.8;88.3) |  |
| With whom the patient lives* |  |  |  |
| Alone | 8 (6.2) | 50.0 (15.7;84.3) | 0.035 |
| Family (with or without a professional caregiver) | 106 (82.2) | 81.1 (72.3;88.1) |  |
| Professional caregiver | 15 (11.6) | 60.0 (32.3;83.7) |  |
| GDS: Global Deterioration Scale, CD: cognitive decline, ChEIs: cholinesterase inhibitors  *statistically significant | | | |
