# Supplementary material for: Burden in caregivers of primary care patients with dementia: influence of neuropsychiatric symptoms according to disease stage (NeDEM project)
Source: BMC Geriatr. 2023 Aug 29;23:525. doi: 10.1186/s12877-023-04234-0 (PMC10463529; doi:10.1186/s12877-023-04234-0)
Supplement: Supplementary file 2 — Supplementary Material 2 [file 12877_2023_4234_MOESM2_ESM.docx]

**Supplement 2.** Relationship between the number of neuropsychiatric symptoms per patient and caregiver burden according to the short ZBI score.

| **Number of symptoms per patient** | **n** | **Short ZBI score** | |
| --- | --- | --- | --- |
|  |  | **Mean** | **(95% CI)** |
| 0 | 2 | 6.5 | (-12.6;25.6) |
| 1 | 3 | 7.7 | (2.5;12.8) |
| 2 | 17 | 10.8 | (9.0;12.6) |
| 3 | 16 | 12.4 | (11.0;13.9) |
| 4 | 18 | 11.1 | (9.1;13.0) |
| 5 | 19 | 12.8 | (10.9;14.8) |
| 6 | 16 | 13.6 | (12.0;15.3) |
| 7 | 14 | 12.5 | (10.6;14.4) |
| 8 | 15 | 13.3 | (10.4;16.3) |
| 9 | 4 | 14.8 | (8.0;21.6) |
| 10 | 5 | 13.6 | (8.4;18.8) |
