# Supplementary material for: Burden in caregivers of primary care patients with dementia: influence of neuropsychiatric symptoms according to disease stage (NeDEM project)
Source: BMC Geriatr. 2023 Aug 29;23:525. doi: 10.1186/s12877-023-04234-0 (PMC10463529; doi:10.1186/s12877-023-04234-0)
Supplement: Supplementary file 3 — Supplementary Material 3 [file 12877_2023_4234_MOESM3_ESM.docx]

**Supplement 3.** Factors associated with caregiver burden (logistic regression models with frequency of neuropsychiatric symptoms and subsyndromes).

|  | **Model 4^1^** | | | | **Model 5^2^** | | |
| --- | --- | --- | --- | --- | --- | --- | --- |
|  | | **OR** | **95% CI** | **p** | **OR** | **95% CI** | **p** |
| **Gender** (Male) | |  |  |  | 4.4 | (1.0; 18.4) | 0.044 |
| **Employment status** (Does not work) | | 5.7 | (1.6; 20.8) | 0.009 | 7.4 | (1.5; 36.5) | 0.013 |
| **Lives with caregiver** (No) | |  |  |  | 7.1 | (1.3; 38.8) | 0.024 |
| **Hours of care** (<8 hours) | | 15.4 | (3.9; 60.2) | 0.000 | 11.8 | (1.9; 73.4) | 0.008 |
| **Patient education** (≥secondary) | | 5.2 | (1.7;15.8) | 0.004 | 21.5 | (4.2; 110.3) | 0.000 |
| **Hyperactivity** **subsyndrome^1^** | | 17.7 | (4.5; 69.6) | 0.000 |  |  |  |
| **Disinhibition frequency^2^** | |  |  |  | 12.7 | (2.5; 65.1) | 0.002 |
| **Irritability frequency^2^** | |  |  |  | 3.0 | (0.9; 9.7) | 0.071 |
| **Aberrant motor activity frequency^2^** | |  |  |  | 7.5 | (1.6; 33.8) | 0.009 |

^1^ logistic regression model adjusted for the frequency of neuropsychiatric subsyndromes (-2LL 98.080)

^2^ logistic regression model adjusted for the frequency of neuropsychiatric symptoms (-2LL 80.542)
