# Supplementary material for: Burden in caregivers of primary care patients with dementia: influence of neuropsychiatric symptoms according to disease stage (NeDEM project)
Source: BMC Geriatr. 2023 Aug 29;23:525. doi: 10.1186/s12877-023-04234-0 (PMC10463529; doi:10.1186/s12877-023-04234-0)
Supplement: Supplementary file 4 — Supplementary Material 4 [file 12877_2023_4234_MOESM4_ESM.docx]

**Supplement 4.** NPI-D score and classification of caregiver distress due to neuropsychiatric symptoms according to the Kaufer model (25).

| **Neuropsychiatric symptoms** | **Distress** | **Low distress (NPI-D 0-1)** | | **Medium distress (NPI-D 2-3)** | | **High distress (NPI-D 4-5)** | |
| --- | --- | --- | --- | --- | --- | --- | --- |
|  | Mean (SD) | n | % (95% CI) | n | % (95% CI) | n | % (95% CI) |
| Elation/euphoria | 1.1 (0.3) | 119 | 92.2 (86.2;96.2) | 9 | 7.0 (3,2;12.8) | 1 | 0.8 (0.0;4,2) |
| Aberrant motor behaviour | 1.3 (0.6) | 102 | 79.0 (71.0;85.7) | 17 | 13.2 (7.9;20.3) | 10 | 7.8 (3.8;13.8) |
| Appetite/eating behaviour | 1.3 (0.6) | 96 | 74.4 (66.0;81.7) | 25 | 19.4 (13.0;27.3) | 8 | 6.2 (2.7;11.9) |
| Disinhibition | 1.4 (0.6) | 94 | 72.9 (64.4;80.3) | 24 | 18.6 (12.3;26.4) | 11 | 8.5 (4.3;14.7) |
| Hallucinations | 1.4 (0.6) | 94 | 72.9 (64.4;80.3) | 24 | 18.6 (12.3;26.4) | 11 | 8.5 (4.3;14.7) |
| Delusions | 1.4 (0.7) | 86 | 66.7 (57.8;74.7) | 30 | 23.2 (16.3;31.5) | 13 | 10.1 (5.5;16.6) |
| Depression/dysphoria | 1.4 (0.7) | 86 | 66.7 (57.8;74.7) | 29 | 22.5 (15.6;30.7) | 14 | 10.8 (6.1;17.5) |
| Anxiety | 1.4 (0.6) | 84 | 65.1 (56.2;73.3) | 35 | 27.1 (19.7;35.7) | 10 | 7.8 (3.8;13.8) |
| Irritability/lability | 1.4 (0.6) | 84 | 65.1 (56.2;73.3) | 34 | 26.4 (19.0;34.8) | 11 | 8.5 (4.3;14.7) |
| Sleep behaviour | 1.5 (0.7) | 83 | 64.3 (55,4;72.6) | 31 | 24.1 (17.0;32.3) | 15 | 11.6 (6.7;18.5) |
| Apathy/indifference | 1.6 (0.7) | 69 | 53.5 (44.5;62.3) | 42 | 32.5 (24.6;41.4) | 18 | 14.0 (8,5;21.2) |
| Agitation/aggression | 1.7 (0.8) | 65 | 50.4 (41.5;59.3) | 35 | 27.1 (19.7;35.7) | 29 | 22.5 (15.6;30.7) |
