# Supplementary material for: Burden in caregivers of primary care patients with dementia: influence of neuropsychiatric symptoms according to disease stage (NeDEM project)
Source: BMC Geriatr. 2023 Aug 29;23:525. doi: 10.1186/s12877-023-04234-0 (PMC10463529; doi:10.1186/s12877-023-04234-0)
Supplement: Supplementary file 5 — Supplementary Material 5 [file 12877_2023_4234_MOESM5_ESM.docx]

**Supplement 5.** Caregiver distress related to neuropsychiatric symptoms (NPI-D score) at each stage of dementia (GDS).

| **Neuropsychiatric symptoms and subsyndromes** | **GDS Stage** | | | | |
| --- | --- | --- | --- | --- | --- |
| **Neuropsychiatric symptoms** | **GDS3** | **GDS4** | **GDS5** | **GDS6** | **GDS7** |
|  | Mean (95% CI) | | | | |
| Disinhibition | 0.4 (-0.5;1.3) | 1.1 (0.7;1.6) | 1.0 (0.6;1.5) | 0.7 (0.2;1.3) | 0.5 (-0.3;1.2) |
| Irritability/lability | 1.1 (0.2;2.1) | 1.3 (0.9;1.8) | 1.2 (0.8;1.7) | 0.8 (0.3;1.3) | 0.5 (-0.1;1.1) |
| Agitation/aggression | 1.6 (-0.1;3.4) | 1.7 (1.1;2.4) | 2.1 (1.5;2.7) | 1.6 (0.9;2.4) | 1.3 (0.1;2.5) |
| Aberrant motor behaviour | 0.1 (-0.2;0.4) | 0.6 (0.2;0.9) | 1.2 (0.7;1.8) | 0.6 (0.1;1.2) | 0.4 (0.0;0.8) |
| Elation/euphoria | 0.6 (-0.4;1.6) | 0.3 (0.0;0.5) | 0.2 (0.0;0.3) | 0.2 (-0.1;0.4) | 0.3 (-0.4;1.0) |
| Apathy/indifference | 1.9 (0.4;3.3) | 1.3 (0.9;1.8) | 1.8 (1.3;2.3) | 1.7 (1.0;2.4) | 1.5 (0.5;2.5) |
| Appetite/eating behaviour | 0.1 (-0.2;0.4) | 0.6 (0.2;1.0) | 1.0 (0.5;1.5) | 1.0 (0.4;1.6) | 0.5 (-0.1;1.2) |
| Delusions | 0.8 (-0.4;1.9) | 0.8 (0.4;1.2) | 1.1 (0.6;1.7) | 1.3 (0.7;1.8) | 1.3 (0.0;2.6) |
| Hallucinations | 0.3 (-0.3; 0.8) | 0.7 (0.3;1.0) | 0.7 (0.3;1.0) | 1.3 (0.7;1.9) | 1.6 (0.3;2.9) |
| Sleep behaviour | 1.3 (0.1;2.4) | 0.8 (0.3;1.3) | 1.1 (0.6;1.6) | 1.6 (0.9;2.3) | 1.6 (0.5;2.7) |
| Depression/dysphoria | 1.9 (0.4;3.3) | 1.1 (0.7;1.5) | 1.2 (0.7;1.7) | 0.8 (0.3;1.4) | 0.9 (-0.1;2.0) |
| Anxiety | 0.8 (0.0;1.5) | 1.1 (0.6;1.5) | 1.1 (0.7;1.6) | 1.0 (0.4;1.6) | 1.4 (0.3;2.5) |
| **Subsyndromes** |  | | | | |
|  | Mean (95% CI) | | | | |
| Hyperactivity | 3.9 (1.5;6.2) | 5.0 (3.7;6.4) | 5.8 (4.3;7.3) | 4.0 (2.7;5.2) | 3.0 (0.4;5.6) |
| Apathy | 2.0 (0.6;3.4) | 1.9 (1.2;2.6) | 2.8 (2.1;3.5) | 2.6 (1.8;3.5) | 2.1 (0.9;3.3) |
| Psychosis | 2.3 (-0.2;4.7) | 2.3 (1.5;3.2) | 2.9 (1.9;4.0) | 4.2 (2.8;5.6) | 4.5 (1.5;7.6) |
| Affective | 2.6 (0.8;4.4) | 2.2 (1.4;2.9) | 2.4 (1.5;3.2) | 1.8 (1.0;2.6) | 2.3 (0.5;4.1) |

GDS: Global Deterioration Scale
